# Non-fatal self-poisoning in Sri Lanka: associated triggers and motivations

**DOI:** 10.1186/s12889-015-2435-5

**Published:** 2015-11-24

**Authors:** Thilini Rajapakse, Kathleen Margaret Griffiths, Helen Christensen, Sue Cotton

**Affiliations:** Department of Psychiatry, Faculty of Medicine, University of Peradeniya, Peradeniya, Sri Lanka; Centre for Mental Health Research, The Australian National University, Building 63, Canberra, ACT 0200 Australia; Black Dog Institute, University of New South Wales, Hospital Road, Randwich, NSW 2013 Australia; Centre for Youth Mental Health, University of Melbourne, 35, Poplar Road, Parkville, VIC 3052 Australia

## Abstract

**Background:**

Attempted or non-fatal self-poisoning is common in Sri Lanka. To date, most preventive strategies have focused on limitation of access to toxic pesticides, which has reduced the rates of *fatal* self-poisoning. However the ongoing phenomenon of *non-fatal* self-poisoning indicates the need for exploration of alternate preventive strategies. Self-poisoning in Sri Lanka has been described as impulsive, with little premeditation, but the motivations associated with this act have not been studied in depth. This research describes the triggers and motivations associated with non-fatal self-poisoning in Sri Lanka. It is anticipated that the findings would help guide future preventive strategies.

**Methods:**

Two studies were carried out, at Teaching Hospital Peradeniya, Sri Lanka, each using a different methodology – Study 1 consisted of qualitative semi-structured interviews, and Study 2 was a cross sectional survey. Both studies were conducted among those who had recently attempted self-poisoning, and explored associated triggers and motivations associated with the act of self-poisoning. There was no overlap between participants of the two studies.

**Results:**

A total of 24 persons participated in the semi-structured interviews (Study 1), and 921 took part in the cross-sectional survey (Study 2). Interpersonal conflict was the most common trigger prior to the act of non-fatal self-poisoning. A mixture of motivations was associated with the act of self-poisoning, including intent to die, to escape, and difficulty tolerating distress associated with interpersonal conflict.

**Conclusions:**

Development of interpersonal skills and interpersonal problem solving skills, particularly in adolescents and young people, emerges as a key primary preventive strategy. Further, there is value in exploring and helping people to develop more adaptive strategies to cope with emotional distress associated with interpersonal conflict. While distress tolerance and interpersonal skill training strategies used in the West may be considered, it is also important to adapt and develop strategies suited to the local cultural background. Further research is needed to develop and evaluate such strategies, and findings may have implications not only to Sri Lanka but also for other countries in South Asia.

## Background

The rate of completed suicides in Sri Lanka has fallen since 1995 [1], but hospital admissions for non-fatal or attempted self-poisoning have increased in recent years [2, 3]. Studies from Southern Sri Lanka have reported intentional self-poisoning rates of 315/100,000 [4], which is comparable to the highest attempted suicide rates reported by the WHO/EURO para-suicide study [5]. Non-fatal self-poisoning is associated with considerable economic cost to the country, as well as personal suffering to the individual. One study estimated that the Sri Lankan government expended US $ 866,304 on treatment of all self-poisonings for the year 2004 [6]. Previous research indicates that non-fatal self-poisoning in Sri Lanka is a phenomenon affecting mostly young adults of both sexes [7, 8], and similar to elsewhere in South Asia, it is often associated with a recent interpersonal conflict [7, 9]. Interpersonal conflict often appears to precipitate the act [7]. However, the motives or reasons for the act, are less clearly described, and may be more difficult to delineate.

In the past, pesticides were the substances most commonly used for self-poisoning in Sri Lanka [10], and interventions to reduce suicide and self-poisoning in in this country have focused primarily on limiting access to lethal pesticides [1, 11]; the significant reduction in completed suicide rates since 1995 has been attributed largely to these ongoing measures [1]. Despite this reduction in completed suicides, rates of *attempted or non-fatal* self-poisoning have been increasing [2, 3]. Broader strategies are now needed to address these growing rates of *non-fatal* self-poisoning in Sri Lanka [3]. This requires better understanding of the motives of individuals who self-harm. Thus, the objectives of this study were to identify the triggers and motivations associated with the act of non-fatal self-poisoning. Minimization of non-fatal self-poisoning is a challenging task but better understanding of the motivations underlying these acts is likely to point the way towards more specific preventive strategies.

## Methods

### Overview of methodology

The motives associated with the act of non-fatal self-poisoning were explored using two studies each employing a different methodology.

In the first study (Study 1), 24 persons who had recently survived an act of self-poisoning were invited to participate in semi-structured qualitative interviews. During these interviews the motivations associated with the acts of self-poisoning were explored in depth. The findings were then used to develop a list of motivations that could be used in a larger, second study.

The second study (Study 2), was a quantitative study, carried out as part of a large cross-sectional survey of people who had survived an act of self-poisoning. Survey participants were invited to report on their motivations for their act of self-poisoning, by perusing the list of motivations derived from Study 1 and indicating whether these motivations applied to them. Further details, and gender differences in non-fatal self-poisoning in Sri Lanka, including a comparison of triggers associated with non-fatal self-poisoning in males and females, have been published previously [12].

Non-fatal self-poisoning was defined as intentional ingestion of a toxic substance or of a medication in excess of its prescribed dosage, with a non-fatal outcome.

Ethical clearance for the studies was obtained from the Faculty of Medicine, University of Peradeniya, Sri Lanka, and the Human Research Ethics Committee of the Australian National University.

### Participants and setting

For both studies, the research was carried out among those who were aged 14 years or older, who had been admitted to the Toxicology Unit, Teaching Hospital Peradeniya, Sri Lanka, for medical management of a recent act of non-fatal self-poisoning. Potential participants were excluded if they could not converse in either Sinhala or English. There was no overlap in the participants for Study 1 and Study 2.

### Study 1 – Semi-structured interviews of those who had recently carried out an act of non-fatal self-poisoning

Persons admitted to the toxicology unit (according to the admission register) on days when the researcher went to the unit, who met inclusion criteria, were invited to participate in the study. Those who gave written informed consent were included in the study. Thus, this was a non-random, non-consecutive sample. Recruitment took place during June-July 2011.

Each participant was interviewed in Sinhalese by the researcher, a specialist in psychiatry, in a confidential setting, within a week of the non-fatal self-poisoning act and prior to discharge from hospital. The semi-structured interview explored the following main themes: events leading up to the non-fatal self-poisoning act, and motivations underlying the act.

At the start of the interview, the participants were encouraged to describe what happened in response to open-ended questions such as “Could you tell me what happened?/What brought you into hospital?” As the interview progressed questions were designed to elicit intentions associated with the self-poisoning act (e.g., “What were your thoughts when you took the tablets/poison?”) followed by more specific questions if required to delineate the strength and nature of the suicidal ideation and other motivations underlying the act (e.g., Did you intend to die when you took the poison?).

The presence of depression or alcohol use disorders was also ascertained through a clinical interview based on ICD10 diagnostic criteria. All interviews were audio-taped, and lasted approximately 45-60 min.

#### Scoring and categorization

The audiotaped interviews were transcribed and translated from Sinhala into English. The transcripts were then analyzed independently by two assessors, one of whom was the first author. The second reviewer was also a doctor, with undergraduate training in psychiatry. Prior to examining the transcripts, both assessors discussed and agreed upon the methods of carrying out the analysis. During the analysis, statements about the immediate triggers and intentions associated with the self-poisoning attempt were extracted, and then itemized in a data extraction sheet, to ensure uniformity. The extracted data were then analyzed for common themes. The two assessors thereafter compared and discussed their findings, and the final categorization based on the consensus findings of both assessors.

### Study 2 - Exploration of motives via the cross sectional survey

As described above, a list of motivations generated after thematic analysis of the semi-structured interviews was administered in the form of a cross-sectional survey. The participants were individuals who had been admitted to the Toxicology Unit of Teaching Hospital Peradeniya, for medical management of self-poisoning, over a consecutive 14-month period. Participants in the cross-sectional survey were invited to peruse a list of possible motivations, and to tick one or more (if any) motivations which they thought were applicable to themselves, at the time that they attempted self-poisoning.

A total of 1334 persons met eligibility criteria to be included in the cross-sectional survey, of whom 9.1 % (*n* = 121) refused consent, and 19.8 % (*n* = 264) could not be included because they either left hospital before the interviews could be conducted, or they were too physically unwell to participate. Therefore a total of 949 participants took part in the survey, of whom 921 (97.0 %) completed the questionnaire regarding motivations associated with the non-fatal self-poisoning act.

## Results

### Study 1 – Semi-structured interviews: Self-reported triggers, and motives associated with the act of non-fatal self-poisoning

The characteristics of the 24 participants and details of the self-poisoning act are given in Table 1. Over 50 % of participants were female, and the median age was 30 years. Pharmaceutical drugs were the most commonly ingested substance, followed by pesticide ingestion. In a majority (>80 %) the act of self-poisoning was premeditated for less than one day, and about a quarter of the participants had a previous history of suicide attempts. At the time of interview, one-third of the participants (33 %, *n* = 8) met ICD 10 diagnostic criteria for depression, and almost one third (29 %, *n* = 7) had an alcohol use disorder (either dependency or harmful use); all of the latter were males. Content analysis of the interviews yielded interpersonal conflict as a main theme associated with the act of non-fatal self-poisoning; over 88 % of participants (*n* = 21) identified a recent interpersonal conflict as an acute stressor leading up to the non-fatal self-poisoning act. In most participants, the conflict was with a close family member (parent, child, sibling or spouse) or with a partner. Only in two instances was the conflict with non-relations (neighbor, school teacher). In all participants who reported an interpersonal trigger, the act of self-poisoning occurred within 24 h of the interpersonal conflict. Three interviewees only did not describe interpersonal conflict as a trigger prior to the act of self-poisoning. These three participants did not describe specific reasons for attempting self-poisoning, but appeared to have carried out the act in the context of agitation associated with a relapse of psychiatric illness – two were experiencing a relapse of schizophrenia, and third had been experiencing symptoms of acute alcohol withdrawal at the time of the act.

**Table 1:** Study 1 – Semi-structured interviews: participant characteristics and details of the non-fatal self-poisoning act

Variable	Median (min-max)	% (n)
Female		54 (*n =* 13)
Age years	30 (14–85)	
Type of poisons ingested		
● Pharmaceutical drug overdose		42 (*n* = 10)
● Pesticides		33 (*n* = 8)
● Other		21 (*n* = 5)
Interpersonal conflict reported as acute trigger associated with self-poisoning attempt		88 (*n* = 21)
Premeditation ≤ 24 h		92 (*n* = 22)
Poison obtained from home or garden		67 (*n* = 16)
Those with history of previous suicide attempts		25 (*n* = 6)
Depressed (ICD 10 criteria)		33 (*n* = 8)
Alcohol use disorder (harmful use/dependency – ICD 10 criteria)		29 (*n* = 7)^a^
Intoxicated at the time of attempted self-poisoning		25 (*n* = 6)^b^

^a^All males

^b^All males who also met criteria for alcohol use disorder

**Fig. 1:**
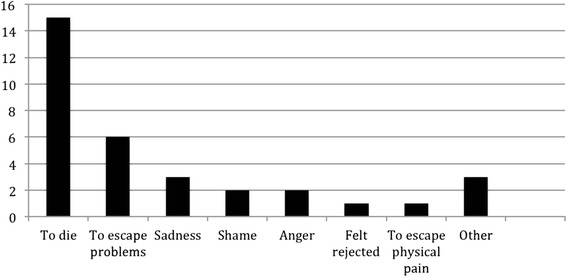
Study 1 - Motivations associated with the act of non-fatal self-poisoning, as described by participants in the semi-qualitative interviews (*n* = 24)

Most interviewed participants reported more than one motive associated with the non-fatal self-poisoning act. These included reported inability to face feelings of shame, distress and emotional pain, physical pain, and anger (Fig. 1). In addition, almost two thirds (63 %, *n* = 15) of the participants reported an intention to die at the time of ingesting the poison. The suicidal intent at the time of the act was expressed in varying ways, such as, “I just wanted to die”, “At that time, I thought I wouldn’t live- I would die”, “I felt sad. I wanted to die, but I didn’t die”, and “I felt there was no point in living.” In contrast the majority of participants (*n* = 16) denied having had ideas of ingesting poison or ideas of suicide *prior* to the interpersonal conflict. Similarly, at the time of the interview, most (71 %, *n* = 17) reported that they were glad to have survived, while the remainder (29 %, *n* = 7) expressed regret or ambivalence at surviving the self-poisoning attempt. A minority (13 %, *n* = 3) expressed ongoing suicidal ideation at the time of interview.

### Study 2 – Cross-sectional survey: Motivations and triggers

Of the 921 participants who took part in the cross sectional survey, 45.5 % (*n* = 419) were males, and the median age was 22 years. The most common method of self-poisoning was by ingestion of a pharmaceutical drug overdose (58.3 %, *n* = 537). Further results of the cross sectional survey have been published elsewhere [12].

More than 65 % (69.7 %, *n* = 642) of participants in the cross-sectional survey described an interpersonal conflict as the immediate stressor prior to the act of non-fatal self-poisoning (Table 2). Of these, interpersonal conflicts with a spouse, parent or regarding a romantic relationship was reported most frequently. The remaining one-third of participants described varying triggers associated with the act of non-fatal self-poisoning, such as intolerable pain symptoms (most often headaches, chest pains or limb pains) and acute stressors related to examinations. A miscellany of triggers, which could not be categorized into groups were included as ‘other’ (16.8 %, *n* = 155).

**Table 2:** Study 2 – findings from the cross-sectional survey (*n* = 921): Triggers associated with the non-fatal self-poisoning act and associations with intention to die

Immediate stressors	% (n)	Association with self-reported intention to die
		*P* value
Argument with spouse	23.4 (222)	.418
Argument with parent	17.7 (168)	<.001
Argument with child	1.9 (18)	.673
Argument with other people	9.4 (89)	.258
Argument and physical assault by husband	1.5 (14)	.210
Conflict regarding romantic relationship (with partner or close family members)	15.4 (131)	.003
Severe financial difficulties	3.8 (35)	.097
Unbearable pain symptoms	4.1 (38)	.008
Exam stress	1.4 (13)	.936
Insomnia or had bad dreams	0.5 (5)	.501
Close family member recently attempted self-poisoning	0.7 (6)	.007
Unwanted pregnancy	0.4 (1)	.365
Other	16.8 (155)	.744
Unable to give a reason	2.8 (26)	.790

Participants of the cross sectional survey reported a mix of motivations associated with the act of non-fatal self-poisoning (Table 3). The most commonly reported motivations or reasons were, a wish to die, a desire to escape an unbearable situation or unbearable thoughts, and an inability to control himself/herself. Those who reported an intention to die were significantly more likely to be male (*χ*^2^(1) = 4.45, *p* = .035), and aged 65 years or older (*χ*^2^(2) = 4.45, *p* = .011). When associations between self-reported intention to die and triggers precipitating the act were examined, recent conflict with parents (*χ*^2^(1) = 13.90, *p* < .001), conflict regarding a romantic relationship (*χ*^2^(1) = 8.89, *p* = .003), suffering from severe pain (*χ*^2^(1) = 7.07, *p* = .008), and having a close family member or friend having attempted self-poisoning recently (*χ*^2^(1) = 7.36, *p* = .007), was significantly associated with an intention to die (Table 2).

**Table 3:** Study 2 - Motivations associated with the non-fatal self-poisoning act – findings from the cross-sectional survey (*n* = 921)

Motivations associated with act	% (n) of participants
To die	53.3 (506)
To escape unbearable thoughts	36.5 (346)
Unable to control himself/herself	32.5 (308)
To escape an unbearable situation	23.4 (222)
To change someone’s mind	14.5 (138)
To make someone sorry	7.4 (70)
Shame - felt unable to face others	6.1 (58)
To show how much I care	2.4 (23)
To show helplessness	1.6 (15)
To find out if anybody cares	1.5 (15)
To sleep for a while	1.4 (13)
To stop being a trouble to others	1.3 (12)
To get help	0.6 (6)
To get revenge	0.6 (6)

## Discussion

Most participants associated the act of non-fatal self-poisoning with a recent interpersonal conflict. This is no surprise – previous studies from Sri Lanka [7, 13] and South Asia [9] have linked self-poisoning behavior to interpersonal conflicts. Interpersonal conflict could be described as a proximal trigger, closely linked to the act of non-fatal self-poisoning and the motivations associated with it. Similarly, interpersonal conflict has been commonly associated with attempted suicide in other parts of Asia, as well as in the West [14–17]. While interpersonal conflict was the most commonly reported trigger, a smaller proportion of participants also described the act of self-poisoning being associated with other triggers, such as severe financial stressors, and unbearable pain symptoms. Interestingly, severe pain symptoms were also significantly associated with a self-reported intent to die. One possible explanation is that the reported somatic pain symptoms may reflect an underlying ‘hidden’ depression, which contributed towards the suicidal intent [18].

Despite differences in culture and background, the motives or *intentions* for non-fatal self-poisoning, described by the participants in these two studies are strikingly similar to motives described in the Western literature - namely a desire to escape or a wish to die [19, 20].

The findings of this research provide additional insight into the processes involved. In particular, a difficulty *tolerating* distress associated with interpersonal conflict emerged as a key factor driving people towards self-poisoning behaviour. For instance, participants of Study 1 described in detail the difficulty in *tolerating* emotional pain following interpersonal conflict, for example - “*I felt very hurt*” (after conflict with daughter); and “*I felt very alone*” (after conflict with mother-in-law and perceived lack of support from husband). A theme of acute distress, and inability to cope with this emotional state emerged clearly – one young female articulated this as, “*I couldn’t bear it. There was nothing else I could think of to do (other than taking poison)*” (after conflict with mother). In Study 2, about one-third of participants described ‘inability to control himself/herself’ at times of emotional distress as contributing towards this act.

Among the semi-structured interviews (Study 1), a 24-year old male participant who drank poison said he had done so because he felt angry and distressed after his mother objected to his choice of girlfriend, but denied having had overt confrontation with his mother about the issue. Self-harm as a means of communicating or enacting anger has been reported by previous Sri Lankan studies [21]. The hierarchical nature of Sri Lankan society where deference to elders is encouraged, may contribute to such situations [22]. Some participants, particularly those who were older, also reported feelings of shame after interpersonal disputes, e.g., - a middle aged male participant stated: “*I felt very ashamed*” (after a dispute with his son about alcohol misuse). The overall emerging feature in the study was emotional distress associated with an interpersonal conflict, and reported inability to deal with that distress.

The majority of participants of the in both Study 1 and Study 2 reported an intention to die at the time of attempting self-poisoning. This finding is consistent with that of a previous Sri Lankan study reported by Hettiarachchi et al. [7]. Males and older participants were significantly more likely to report an intention to die – this is in keeping with international findings that increasing suicidal intention is associated with older age and male gender [23, 24].

In most instances, the desire to die was expressed in the context of acute distress associated with interpersonal conflict. In keeping with this, by time of interview (within one week of the act of non-fatal self-poisoning), the majority of participants of the semi-structured interviews reported no current suicidal ideation, and was glad to have survived. Likewise, the duration of premeditation associated with the act of non-fatal self-poisoning was short. Most of the acts of non-fatal self-poisoning occurred within 24 h of the interpersonal conflict, similar to previous Sri Lankan studies [25, 26].

While the desire to escape, or to die, emerge as key themes, overall motivations often appeared mixed and complex. Although not the most commonly reported motivations, many participants in the cross-sectional survey subscribed to motives such as *to change someone's mind, to make someone sorry, to show how much I care, to show helplessness* – which appear to be acts of communication with a significant other. Further, the distressing emotions described by the participants, such as sadness, anger, or shame are all likely to have contributed towards self-poisoning behaviour.

Notably, none of the participants in either study identified depression as being associated with the non-fatal self-poisoning act. This was despite the fact that about one third of the interview participants (Study 1) were clinically depressed at interview. This is in contrast to findings from the West [27], where survivors have described their self-harm attempts as being associated with depression. This difference in the way persons interpret their experiences may be partly due to the fact that in Sri Lanka, as in other South Asian cultures, the symptoms of depression are often not conceptualized as a disorder [28], and indeed there is no colloquial term for depression in the Sinhala language.

### Limitations

The semi-structured interviews were limited by the small sample size, but a strength of the study was the structured, detailed nature of the interviews, and that relevant information was extracted systematically by use of audio-taping, transcribing, data extraction sheets and dual coding of the intervention strategies. Although the data was assessed by two raters, inter-rater reliability was not formally assessed which is a limitation. The assessors discussed and agreed upon the method of assessment prior to analysis, but there was no formal training prior to the analysis, which also may have been a limitation. However, the qualitative nature of this study added depth and detail to the information gathered through the quantitative survey. The retrospective recall of events by participants in both the interviews and cross-sectional survey is also a limitation, since this method may have led to a recall bias, although efforts were made to minimize this by conducting the interviews within one week of the non-fatal self-poisoning act. Further, due to restriction of numbers, it was not possible to undertake further analysis of possible associations between different types of triggers and motivations, and this is a further limitation of the study.

## Conclusions

Two main findings emerge from the two studies reported here: (i) interpersonal conflict is a proximal trigger associated with non-fatal self-poisoning, in most instances; and (ii) a mixture of motivations is associated with the act, including a desire to die, a desire to escape, and difficulty tolerating distressing emotion associated with interpersonal conflict. The intention to die, although commonly reported, appears short lived and associated with the emotional distress.

These findings have important implications for the prevention of self-poisoning in Sri Lanka. Development of interpersonal skills and interpersonal problem solving skills, particularly in adolescents and young people, emerges as a key preventive strategy. For example, interventions such as grass-root level community and youth programs to help people develop ways of dealing with interpersonal stress warrant further exploration, and such programs have shown promise internationally [29, 30]. However it is also important to tailor these programs to suit the local socio-cultural framework. In particularly, factors such as the collectivistic rather than individualistic nature of society in Sri Lanka, and the hierarchical framework where overt confrontation is discouraged – which in itself might be contributing towards the interpersonal stress – should be taken into account when conceptualizing such programs [31]. Another worthwhile avenue to explore is whether the internet could be used to engage young people – social media such as Facebook is now popular among urban Sri Lankan youth, and online interventions may provide a way of delivering training in interpersonal skills. Online interventions in the West have demonstrated efficacy in reducing symptoms of depression [32].

Difficulty in coping with acute distress related to interpersonal conflict, was another key aspect that emerged from this study. While distress tolerance strategies have been explored in the West [33], this is a little researched area in Sri Lanka, and indeed much of South Asia. There is value in exploring and helping people to develop more adaptive strategies to cope with emotional distress associated with interpersonal conflict, and once again, it is important to adapt and develop strategies suited to the local cultural background. Development of such programs requires further research and evaluation, and findings may have implications not only to Sri Lanka but also for other countries in South Asia.
